# Assessment of patient-centered outcomes (PROs) in inflammatory bowel disease (IBD): a multicenter survey preceding a cross-disciplinary (functional) consensus

**DOI:** 10.1186/s12955-020-01489-8

**Published:** 2020-07-20

**Authors:** Xiaohan Yan, Yuqi Qiao, Jinglu Tong, Ren Mao, Jie Liang, Cuicui Lv, Yueying Chen, Yiyan Wang, Jun Shen, the Young Investigators for the study of Inflammatory Bowel Diseases (YIIBD)

**Affiliations:** 1 Division of Gastroenterology and Hepatology, Key Laboratory of Gastroenterology and Hepatology, Ministry of Health, Inflammatory Bowel Disease Research Center; Renji Hospital, School of Medicine, Shanghai Jiao Tong University; Shanghai Institute of Digestive Disease, 160# Pu Jian Ave, Shanghai, 200127 China; 2 Endoscopy Center, Shanghai East Hospital, Tongji University School of Medicine, Shanghai, 200120 China; 3 Department of Gastroenterology, the First Affiliated Hospital, Sun Yat-sen University, No.58, Zhong Shan Er Lu, Guangzhou, 510080 China; 4 Department of Gastroenterology, Xijing Hospital, Air Force Medical University, Chang Le Xi Lu, Xi’an, 710032 China; 5 Department of Gastroenterology, the Third Hospital, Liao Cheng, China; 6 Department of Gastroenterology, Zhong Shan Hospital, the Xiamen University, Xiamen, China; 7 Department of statistics, University of Leeds, Leeds, England

**Keywords:** Patient-centered outcome (PRO), Inflammatory bowel disease (IBD), Quality of care (QoC)

## Abstract

**Background:**

With a shift in the healthcare paradigm towards a more patient-centered approach, data on inflammatory bowel disease (IBD) needs to be further explored. This study aimed to determine patient perspectives on the effect of IBD and features of patients with lower satisfaction level and compare patient and physician perception of IBD-related Quality of Care (QoC).

**Methods:**

A previously developed pre-standardised set of questions regarding patient-centered outcome (PRO) measures for IBD, comprising 36 items, was administered in five centers, and a concomitant questionnaire for specialised physicians was adapted and administered.

**Results:**

Overall, 1005 patients with IBD met the inclusion criteria. Sixty-five questionnaires were administered to specialised physicians. Both patients and physicians perceived the IBD-related QoC as being satisfactory. Furthermore, this study revealed areas of shortcomings where it comes to patient perceptions. Female sex and the presence of negatively impacting disease characteristics (presence of significant pain or discomfort, lack of energy, feeling fatigued most of the time, experiencing anxiety or depression in the last 2 weeks) were associated with lower satisfaction levels.

**Conclusions:**

Our findings can be used in establishing strategies aimed at improving patient QoC and defining strategic priorities. These data can aid in improving the communication of the pressing needs of IBD patients, to both the public payers and health authorities.

## Background

Inflammatory bowel disease (IBD), comprising Crohn’s disease (CD), ulcerative colitis (UC) and IBD unclassified (IBD-U), is a chronic immune disease that frequently emerge early in life and requires chronic health care. The wide spectrum of IBD disease severity and frequency of complication occurrence impair patient physical and mental health, quality of life (QoL), and social functioning, resulting in the need for chronic intensive disease monitoring, regular outpatient appointments, hospitalization, and surgery [1]. The prevalence of IBD is increasing globally, with the incidence rising particularly rapidly in Asia [1], accompanied by increasing rates of hospitalization, cumulative surgery, reoperation, and permanent work disability [2, 3]. All these lead to high costs to patients and the healthcare system [4]. Quality of care (QoC) is defined as “the degree to which health services for individuals and populations increase the likelihood of desired health outcomes and are consistent with current professional knowledge” [5]; subsequently, the provision of advanced QoC reduces the per capita costs of healthcare [6]. In some studies, QoC was related to both healthcare provider use of technology and interpersonal skills, which depend greatly on the quality of their communication with patients [7, 8]. The evaluation of QoC consists of the quality of the services provided and patient perception of services received. There is always a gap between physician and patient perception of QoC [6, 9, 10].

Healthcare is transitioning towards a value-based system [11, 12]. The acceleration of outcome measurement can unlock the potential of value-based healthcare in driving improvements [13]. Alongside traditional biomedical measures, patient-centered outcome measures are increasingly being used for the evaluation of the management and treatment of different diseases [14–18]. Efforts should be driven towards the application of the patient-centered outcome (PRO) measures in chronic conditions, as the major goals of management are the reversal of disease progression or its arrest, and QoL improvements. Considering the global burden of IBD and its wide-ranging effects on QoL, several attempts have been made aimed at the development of patient-assessed health outcome measures [19]. However, patient-reported outcomes other than those pertaining to disease-related symptoms or QoL are rarely captured in IBD care settings. Therefore, this study was performed with the establishment of the “Standard Set of Patient-centered Outcomes for Inflammatory Bowel Disease – an International, Cross-disciplinary Consensus” as the background survey for the enhancement of actuality basis.

The aims of this study were to assess patient-centered outcomes, including patient perception of QoC, and investigate whether patient-reported outcomes are associated with demographic factors and clinical status. Subsequently, the differences between patient and physician perception of QoC were also determined.

## Methods

### Participants and methods

Patients in this multicenter, observational study were enrolled from three hospitals across different provinces of China, covering a population greater than 50,000,000 people. From March 2016 to November 2017, consecutive inpatients and outpatients with an IBD diagnosis were enrolled.

Inclusion criteria were:

- age at least 18 years;
- verified IBD diagnosis (based on a combination of clinical, laboratory, endoscopic, radiological, and histological findings); and
- ability to understand the questionnaire and provide written informed consent.

Exclusion criteria were:

- follow-up duration at the center shorter than 12 months at the time of enrolment; or
- inclusion in other clinical trials at baseline.

The questionnaire was delivered either on paper or online to each prospective outpatient while they were waiting for their clinic appointment. Most of the patients submitted a completed questionnaire to the investigators while still in the waiting room. Some patients completed the online version of the survey in the waiting room or shortly thereafter. Inpatients received the questionnaire during their hospital stay. IBD specialists were involved in the entire process of questionnaire admisnistration, and subsequently checked all answers for missing values or inconsistencies. Another concomitant electronic questionnaire for physicians was administered to IBD specialists from multiple hospitals across East China via email.

### Questionnaires

A standard set of patient-centered outcome measures for IBD was previously developed by an international working group (*n* = 25), representing patients, patient associations, gastroenterologists, surgeons, specialist nurses, IBD registries and patient-reported outcome measure methodologists [19]. A systematic review of existing literature, registry data, patient focus groups, a series of teleconferences incorporating a modified Delphi process, and open review periods were used to reach a consensus on a minimum set of standard outcome measures and risk adjustment variables. A pre-standardised set was delivered before the final conference.

In this study, we used the simplified Chinese version of a pre-standardised set comprising 36 items. The questionnaire covered four domains:

- Survival and disease control
- Healthcare utilization
- Disutility of care
- Symptoms, function, and QoL

Except for the descriptive items and two open-ended items, the remaining questions were optional. Socio-demographic variables were self-reported by patients and included data on diagnosis, sex, age, age at diagnosis, education level, work, smoking habits, and surgery history. The questionnaire is presented in Additional Table 1 (Addendum).

Another concomitant electronic questionnaire for physicians was adapted from the patient version, as shown in Additional Table 2 (Addendum).

### Data analysis

To investigate the potential association of patient satisfaction with clinical, epidemiological parameters, and patient-centered outcomes, bivariate correlation analyses (using Pearson’s correlation coefficient and Spearman‘s correlation coefficient) was performed. Independent sample t-tests and one-way analysis of variance, as well as a chi-square test were performed to investigate differences in the clinical, epidemiological parameters, and patient-centered outcomes across the different subgroups. All tests were two-sided with a significance level of 5%, and were performed using R version 3.3.3.

## Results

### Characteristics of enrolled participants

Overall, 1005 patients with IBD met the inclusion criteria; 925 of them agreed to participate and provided written informed consent. Finally, 891 of the questionnaires (response rate: 88.7%) for patients were found to be evaluable. Overall, 522 patients (58.6%) had CD, 363 (40.1%) had UC, and six (0.7%) had IBD-U. The socio-demographic and clinical data of the responders, by diagnostic group, are shown in Table 1. In addition, 65 questionnaires were administered to IBD specialists, and 55 were considered evaluable. The average duration of physician involvement in IBD care provision was 7 (range: 3–25 yrs) years. The average percentage of work time involving the provision of care to IBD patients was 12% (range: 6–21%).

**Table 1:** Characteristics of patients responded to the survey

	CD	UC	Total	*P* value (CD vs. UC)
Number of patients, *N* (%)	522 (58.6%)	363 (40.1%)	891	
Female sex, *N* (%)	201 (38.5%)	164 (45.2%)	362 (41%)	0.053
Age, years (range)	37 (18–69)	44 (18–75)	40 (18–75)	0.913
Disease duration since diagnosis, years (range)	4.1 (1–21)	4.5 (1–25)	4.2 (1–25)	0.214
Education level, *N* (%)				
Primary	37 (7.1%)	38 (10.5%)	75 (8.4%)	
Secondary	120 (23%)	92 (25.3%)	214 (24%)	
university	365 (69.9%)	233 (64.2%)	601 (67.5%)	0.238
Current occupation, *N* (%)				
Working (employee)	281 (53.8%)	218 (60.1%)	501 (56.2%)	0.136
Working (self-employed)	55 (10.5%)	52 (14.3%)	109 (12.2%)	
Studying	45 (8.6%)	15 (4.1%)	60 (6.7%)	
Unemployed	36 (6.9%)	11 (3%)	47 (5.3%)	
Long-term sick leave because of IBD	69 (13.2%)	17 (4.7%)	86 (9.7%)	
Long-term sick leave for other reasons	1 (0.2%)	0 (0%)	1 (0.1%)	
Retired early due to IBD	1 (0.2%)	4 (1.1%)	5 (0.6%)	
Retired early for other reasons	13 (2.5%)	14 (3.9%)	27 (3%)	
Retired (because of age)	11 (2.1%)	29 (8%)	42 (4.7%)	
Fulfilling family role as parent or partner	10 (1.9%)	3 (0.8%)	13 (1.5%)	
Smoker, *N* (%)				
Every day or almost every day	39 (7.5%)	39 (10.7%)	80 (9%)	
At least once per week	6 (1.1%)	14 (3.9%)	20 (2.2%)	
At least once per month	2 (0.4%)	5 (1.4%)	7 (0.8%)	
Never	475 (91%)	305 (84%)	784 (88%)	0.174
Abdominal surgery history for IBD, *N* (%)	99 (19%)	92 (25.3%)	191 (21.4%)	0.205

### Patient-reported outcome measures

In the 2 weeks preceding questionnaire administration, 61.7% of patients with CD and 41% of those with UC experienced anxiety or depression due to IBD (*p* = 0.029). Among all the IBD patients with various current occupations, we found that 31 patients with “unemployment” and 58 with “long-term sick leave” shared the highest proportion of feeling anxious or depressed because of his/her IBD. In other words, 86.1% of the unemployed patients and 84.1% of the “long-term sick leave” patients felt anxious or depressed, which is much more frequent than those observed in other current occupations, such as “working (employee)” and “working (self-employed)” and “studying”, as well as “long-term sick leave for other reasons”, “early retirement due to IBD”, “early retirement for other reasons”, “retirement (because of age)” and “fulfillment of family role as parent or partner”. In the 12 months preceding questionnaire administration, the numbers of hospitalizations due to IBD-related symptoms in the CD group and UC group were 1.72 (1.8) and 0.71 (1.6), respectively (*p* = 0.001). The proportion of absenteeism from school or college due to IBD-related symptoms in the CD and UC cohorts were 65.6 (108.9) and 28.5 (76.4) days, respectively (*p* = 0.002). The durations of absence from work in the CD and UC groups were 71.1 (118.2) and 31.7 (84.3) days, respectively (*p* = 0.001). In the 24 months preceding questionnaire administration, the longest continuous durations of steroid use for IBD in the CD and UC groups was 2.1 (11.8) and 0.5 (4.2) months, respectively (*p* = 0.001). Detailed data on the patient-centered outcomes of the responders, by diagnostic group, are shown in Table 2.

**Table 2:** Patient-centered outcome measures

	CD (522)	UC (363)	*P* value
In the last two weeks,			
IBD has been well controlled (Yes/ No/ Not sure)	201/133/188 (38.5%/25.5%/36%)	158/120/85 (43.5%/33.1%/23.4%)	0.173
Current treatment is useful in controlling IBD (Yes/ No/ Not sure/ without any treatment)	211/121/160/30 (40.4%/23.2%/30.7%/5.7%)	118/76/154/15 (32.5%/20.9%/42.4%/4.1%)	0.288
Miss any planned activities because of IBD (Yes/ No/ Not sure)	203/244/75 (38.9%/46.7%/14.4%)	141/158/64 (38.8%/43.5%/17.6%)	0.999
Wake up at night because of symptoms of IBD (Yes/ No/ Not sure)	191/233/98 (36.6%/44.6%/18.8%)	136/190/37 (37.5%/52.3%/10.2%)	0.997
Suffer from significant pain or discomfort (Yes/ No/ Not sure)	223/245/54 (42.7%/46.9%/10.3%)	176/120/67 (48.5%/33.1%/18.5%)	0.375
Feel lacking in energy fatigued for more than half the time (Yes/ No/ Not sure)	319/180/23 (61.1%/34.5%/4.4%)	212/117/34 (58.4%/32.2%/9.4%)	0.349
Feel anxious or depressed because of IBD (Yes/ No/ Not sure)	322/142/58 (61.7%/27.2%/11.1%)	149/130/84 (41%/35.8%/23.1%)	0.029
Need a change to treatment (Yes/ No/ Not sure)	144/173/205 (27.6%/33.1%/39.3%)	121/108/134 (33.3%/29.8%/36.9%)	0.083
In the last 12 months,			
Times of flare-ups	4.9 (11.9)	2.3 (3.7)	0.064
Times of hospitalization because of IBD-related Symptoms	1.72 (1.8)	0.71 (1.6)	0.001
absent from school or college due to IBD-related Symptoms (days)	65.6 (108.9)	28.5 (76.4)	0.002
absent from work due to IBD-related symptoms (days)	71.1 (118.2)	31.7 (84.3)	0.001
unable to do normal activities due to IBD-related Symptoms (days)	32.4 (70.5)	14 (50)	0.071
In the last 24 months, the longest continuous stretch of time of taking steroid tablets for IBD (months)	2.1 (11.8)	0.5 (4.2)	0.001

### Experience of IBD care

The average duration from the time medical care was first sought for symptoms that were finally recognized as IBD-related to IBD diagnosis confirmation was 9.8 (19.8) months. The duration to diagnosis significantly differed between the CD and UC groups (13.3 (24.8) vs. 4.5 (5.8) months, *p* < 0.001), and were positively correlated to age (correlation coefficient = 0.036, *p* < 0.001). There were no significant differences between male and female participants (*p* = 0.194). The average frequency of emergency room presentations for IBD-related symptoms before a definitive diagnosis was reached was 2.6 (5.4) times. The average frequency of emergency room presentation significantly differed between the CD and UC groups (3.4 (6.4) vs. 0.99 (1.4) months, *p* < 0.001). There was no significant difference between male and female participants in this regard (*p* = 0.070). In the whole cohort, the frequency of emergency room presentation was positively correlated to time to diagnosis (correlation coefficient = 0.215, confidence interval [CI]: 0.023–0.506, *p* < 0.001). However, the time to diagnosis was not correlated to age (*p* = 0.294).

In total, 855 patients (96%) consulted gastroenterologists for IBD, 24 patients (2.7%) consulted general surgeons, 82 (9.2%) consulted colorectal surgeons, 13 (1.5%) consulted other specialists, seven (0.8%) consulted specialist nurses, and none of the patients consulted their family doctor. Overall, 615 patients (69%) had a regular IBD review appointment regardless of IBD activity status, whereas 276 patients (31%) did not. There was no significant difference based on sex (*p* = 0.338), education level (*p* = 0.412), and age (*p* = 0.371) in this regard. The average duration of the last IBD consultation was 11.1 (8.3) minutes in the CD group and 12.2 (14.4) minutes in the UC group (*p* = 0.328). There was no significant difference between the sexes (*p* = 0.172) and between patients of different ages (*p* = 0.379). In total, 423 patients (47.5%) felt that the consultation time was sufficient for a satisfactory review of the IBD status, whereas 468 patients (52.5%) did not feel this to be the case. Eight-hundred-and-41 patients (94.4%) received specialist advice at a hospital or clinic during a flare-up. Few patients received information by telephone (11.2%) or email (0.6%). An interesting finding was that 55 patients (29.3%) felt that the number of toilets was sufficient for patients with IBD during hospitalization, whereas 133 patients (70.7%) did not.

### Satisfaction with care

Overall, 295 (56.6%) patients with CD, 220 (60.6%) patients with UC, and 46 (83.6%) specialists felt that the level of communication between patients and health specialists was excellent or very good (CD group vs. UC group: *p* = 0.724; specialists vs. IBD patients: *p* = 0.008). A larger number of specialists than patients believed that the level of communication was good; however, there was no difference between patients with CD and UC in this regard. A total of 347 (66.4%) patients with CD, 276 (76%) patients with UC, and 45 (81.8%) specialists regarded the quality of the IBD care received or provided in the past 12 months as being excellent or very good (CD group vs. UC group: *p* = 0.898; specialists vs. IBD patients: *p* = 0.231). There was no difference between patients with CD and UC, or between the specialists and patients in this regard. Female patients showed a higher tendency to feel that the quality of communication with specialists (*p* = 0.037) and quality of IBD care (*p* = 0.019) was less satisfactory than male patients. However, patients across different age groups (*p* = 0.231), disease duration groups (*p* = 0.307), education level groups (*P* = 0.549), and history of abdominal surgery for IBD groups (*p* = 0.113) did not show significant differences in terms of the level of satisfaction pertaining to communication with specialists. Similarly, patients across different age groups (*p* = 0.275), disease duration groups (*p* = 0.292), education level groups (*p* = 0.687), and history of abdominal surgery for IBD groups (*p* value = 0.202) did not show significant differences in the quality of IBD care. In the bivariate analyses conducted to further evaluate patient-centered outcomes, patients with significant pain or discomfort were less satisfied with the quality of IBD care than those without (*P* = 0.043). Patients who felt fatigued for the majority of the time were less satisfied with the quality of IBD care than those who did not (*p* = 0.028). Patients who had experienced anxiety or depression in the last 2 weeks were less satisfied with the quality of IBD care than those who had not (*p* = 0.003). There was no statistically significant difference in the perception of IBD management (*p* = 0.219).

Of all the topics discussed in the consultations, there were significant differences in regarding medical treatment options (13.4% of the patients in the CD group and 30.9% of those in the UC group felt that this aspect was “not sufficiently discussed,” *p* < 0.001), use of new/experimental treatments (38.7% of the patients in the CD group and 21.5% of those in the UC group felt this aspect was “sufficiently discussed,” whereas 3.1 and 19.6% of those in the CD and UC groups, respectively, felt it was “not sufficiently discussed,” *p* < 0.001). Detailed data on the patient and specialist perception of satisfaction with healthcare are presented in Table 3.

**Table 3:** Patients’ and physicians’ satisfaction with care

	CD (522)	UC (363)	*P* value^1^	Physician (55)	*P* value^2^
IBD management is well coordinated			0.059		0.219
very well coordinated	156 (29.9%)	115 (31.7%)		19 (34.5%)	
fairly well coordinated	269 (51.5%)	198 (54.5%)		28 (50.9%)	
fairly uncoordinated	90 (17.2%)	37 (10.2%)		8 (14.5%)	
completely uncoordinated	7 (1.3%)	13 (3.6%)		0 (0%)	
Communication with health specialists			0.724		0.008
Excellent	77 (14.8%)	71 (19.6%)		8 (14.5%)	
Very good	218 (41.8%)	149 (41%)		38 (69.1%)	
Good	172 (33%)	98 (27%)		8 (14.5%)	
Fair	51 (9.8%)	44 (12.1%)		1 (1.8%)	
Poor	4 (0.8%)	1 (0.3%)		0 (0%)	
Quality of the IBD care received in the past 12 months			0.898		0.231
Excellent	90 (17.2%)	45 (12.4%)		12 (21.8%)	
Very good	257 (49.2%)	231 (63.6%)		33 (60%)	
Good	127 (24.3%)	62 (17.1%)		10 (18.2%)	
Fair	23 (4.4%)	12 (3.3%)		0 (0%)	
Poor	3 (0.6%)	2 (0.6%)		0 (0%)	
Without any IBD care	22 (4.2%)	11 (3%)			
The following topics were sufficiently discussed in consultations?					
Current symptoms (Yes/ No/ No need to discuss/ unaware)	301/98/33/90 (57.7%/18.8%/6.3%/17.2%)	243/51/37/32 (66.9%/14%/10.2%/8.8%)	0.067	49/3/0/3 (89.1%/5.5%/0%/5.5%)	0.001
Medical treatments (Yes/ No/ No need to discuss/ unaware)	341/70/31/80 (65.3%/13.4%/5.9%/15.3%)	93/112/36/22 (53.2%/30.9%/9.9%/6.1%)	0.001	47/3/1/4 (85.5%/5.5%/1.8%/7.3%)	0.001
Surgery (Yes/ No/ No need to discuss/ unaware)	81/35/180/226 (15.5%/6.7%/34.5%/43.3%)	71/21/132/139 (19.6%/5.8%/36.4%/38.3%)	0.441	45/2/1/7 (81.8%/3.6%/1.8%/12.7%)	0.001
New/experimental treatments (Yes/ No/ No need to discuss/ unaware)	202/16/103/201 (38.7%/3.1%/19.7%/38.5%)	78/71/89/125 (21.5%/19.6%/24.5%/34.4%)	0.001	50/2/1/2 (90.9%/3.6%/1.8%/3.6%)	0.001
Nutrition/diet (Yes/ No/ No need to discuss/ unaware)	278/132/6/106 (53.3%/25.3%/1.1%/20.3%)	213/121/5/24 (58.7%/33.3%/1.4%/6.6%)	0.641	50/1/1/3 (90.9%/1.8%/1.8%/5.5%)	0.001
Practical daily living (Yes/ No/ No need to discuss/ unaware)	321/145/27/29 (61.5%/27.8%/5.2%/5.6%)	203/112/14/34 (55.9%/30.9%/3.9%/9.4%)	0.218	41/9/2/3 (74.5%/16.4%/3.6%/5.5%)	0.001
Education/studies (Yes/ No/ No need to discuss/ unaware)	265/167/69/21 (50.8%/32%/13.2%/4%)	162/108/38/55 (44.6%/29.8%/10.5%/15.2%)	0.485	41/7/3/4 (74.5%/12.7%/5.5%/7.3%)	0.001
Employment (Yes/ No/ No need to discuss/ unaware)	221/146/76/79 (42.3%/28%/14.6%/15.1%)	139/112/56/56 (38.3%/30.9%/15.4%/15.4%)	0.069	47/5/1/2 (85.5%/9.1%/1.8%/3.6%)	0.001
Personal relationships (Yes/ No/ No need to discuss/ unaware)	309/104/78/31 (59.2%/19.9%/14.9%/5.9%)	194/82/42/45 (53.4%/22.6%/11.6%/12.4%)	0.374	21/18/5/11 (38.2%/32.7%/9.1%/20%)	0.001
Sexual relationships (Yes/ No/ No need to discuss/ unaware)	218/117/132/55 (41.8%/22.4%/25.3%/10.5%)	143/89/97/34 (39.4%/24.5%/26.7%/9.4%)	0.485	31/15/1/8 (56.4%/27.3%/1.8%/14.5%)	0.001
General lifestyle issues (Yes/ No/ No need to discuss/ unaware)	279/129/61/53 (53.4%/24.7%/11.7%/10.2%)	202/71/39/51 (55.6%/19.6%/10.7%/14%)	0.788	29/19/4/3 (52.7%/34.5%/7.3%/5.5%)	0.429
Main goals or priorities in caring for current condition (Yes/ No/ unaware)	377/102/43 (72.2%/19.5%/8.2%)	276/58/29 (76%/16%/8%)	0.572	49/6/0/0 (89.1%/10.9%/0%/0%)	0.047
Make a plan for carrying out in daily life (Yes/ No/ unaware)	331/105/86 (63.4%/20.1%/16.5%)	218/83/62 (60.1%/22.9%/17.1%)	0.374	39/11/1/4 (70.9%/20%/1.8%/7.3%)	0.042

1: *P* value (CD vs. UC)

2: *P* value (patients vs. physicians)

Using Spearman’s correlation, we found that patient level of satisfaction with the quality of IBD care was correlated with the presence of well-coordinated IBD management (correlation coefficient = 0.18, CI: 0.061–0.703, *p* = 0.009) and satisfaction with the level of communication with health specialists (correlation coefficient = 0.457, CI: 0.329–0.692, *p* < 0.001), but was not correlated to adequacy in the number of toilets during time of IBD hospitalisation (correlation coefficient = 0.066, CI: − 0.075 – 0.225, *p* = 0.334).

The topics that the patients with CD regarded as being insufficiently discussed in the consultations (over 10%) in the order of “highest to lowest” were: education/studies, employment, practical daily living, nutrition/diet, general lifestyle issues, sexual relationships, formulation of a plan for daily life, personal relationships, main goals or priorities in caring for the current condition, current symptoms, and medical treatments. The corresponding topics in the UC group ranked from highest to lowest were: nutrition/diet, employment, practical daily living, medical treatments, education/studies, sexual relationships, formulation of a plan for daily life, personal relationships, general lifestyle issues, new/experimental treatments, main goals or priorities in caring for the current condition, and current symptoms (Table 3).

### Analysis of the open-ended items

The two open-ended questions were: “What is the best thing about the care you currently receive?” and “What is the care that you would most like to be changed or improved?” The answers were as follows:

- fewer expenses and better coverage (for drugs such as infliximab) by health insurance;
- provision of more efficient and better-coordinated management, shorter waiting times, and easier access to the purchase of medicines;
- better disease control and fewer hospital visits;
- better level of communication and easy access (such as telephone and Wechat), with a greater degree of information on diets and medicines, as well as disease control (more public lectures and popular science articles);
- demand for the combined use of traditional Chinese medicine with Western medicine;
- better inpatient facilities and a larger number of IBD healthcare providers.

## Discussion

In this study, we found that both patients and physicians perceived the IBD-related QoC as being satisfactory. Furthermore, we uncovered shortcomings according to the patient perception. Female sex and the presence of certain negatively impacting disease characteristics were associated with lower satisfaction levels.

The importance of the systematic tracking and reporting of patient-centered outcomes is gradually being realized. Through the comparison of meaningful patient outcomes, providers can adopt strategies aimed atachieving the best “value” for participating stakeholders; this is defined as value-driven care [19]. The present study was devoted to the pursuit of this aim. When the provision of care is driven by outcomes, remission rates go up [20]. The US Food and Drug Administration (FDA) has advocated the routine inclusion of patient-reported outcomes as co-primary endpoints in clinical trials [21]. Although QoL improvement is a commonly used secondary endpoint in clinical trials, it was the primary endpoint in only one trial focusing on IBD [22] and is rarely captured in routine practice.

In this study, 61.7% of the patients with CD and 41% of those with UC experienced anxiety or depression due to IBD in the 2 weeks preceding questionnaire administration. Unemployment and taking long-term sick leave due to IBD ranked the highest in terms of the causes of anxiety and depression, compared to other current occupations. A previous study showed that absence from work and the presence of active disease were the two main factors associated with a reduced health-related QoL (HRQoL) [23]. Another study proved that absence from work, number of physician visits, and the proportion of procedures undergone were related to an impaired HRQoL [24]. Thus, a greater degree of attention should be paid to those with unemployment and long-term sick leave. Previous studies have shown that IBD is associated with a high prevalence of anxiety and depression. Psychological therapies and cognitive behavioral therapy may be beneficial in decreasing the levels of anxiety and depression in IBD [25–27].

None of the patients consulted family doctor. It should be mentioned that there are very few family doctors or general practitioners in China, and that most patients consult specialists.

There has been a tendency for patient perception of the quality of healthcare to be ignored as they are not adequately aware of what constitutes high care quality. As of 2018, no consensus on patient satisfaction or Quality of Care (QoC) measurement had been reached [28]. Previous studies focusing on patient satisfaction with IBD care presented conflicting results in terms of the factors that affect their QoC-related perception [29–31]. A judgment of low quality may have resulted as a consequence of extremely high expectations or the provision of a very poor QoC [32]. In addition, it cannot be directly concluded that the conflicting results reflects the diversity of various care centers and geographical location, since this could be explained by methodological differences or bias caused by the selection of participants. In this multicenter study, only 5% of the patients with CD and 3.9% of those with UC, and 0% of the physicians stated that they were dissatisfied with the quality of IBD care, consistent with previous studies [33]. Therefore, the patients with IBD were satisfied with the QoC provided in the included hospitals. Since each investigated hospital has an IBD care center consisting of an IBD-specialized inpatient and outpatient department and a multidisciplinary team, these measures are inferred to be essential for the improvement of the level of satisfaction with the quality of IBD care. A previous study has shown that even hospitals with a minimal IBD service performed better in terms of many process and outcome measures. Hospitals with a partial IBD service showed a greater degree of adherence to the standards for safety monitoring of biologic (89% vs. 59%) and immunosuppressive drugs (79% vs. 55%), and a rate of admission via an emergency department that was 22% lower than that in hospitals without such a service [34]. Furthermore, the introduction of an IBD nurse is also essential, which leads to significant cost savings and better QoC, through the reallocation of physician time resources and reductions in the hospitalization rates [35]. As is well-known, the incidence of IBD has increased rapidly in Asia in recent years, especially in countries undergoing rapid industrialization [36, 37]. Our data on the IBD-related QoC may aid local governments in more effectively allocating resources for accelerating the construction of specialized and dedicated IBD care centers [38].

In our study, patient satisfaction with the quality of IBD care was related to the satisfaction with the level of communication with specialists. Patient-physician communication is vital in ensuring patient commitment and involvement in long-term disease self-management [38], and influences complementary and alternative therapies [39]. In medical care, communication is highly associated with better patient adherence to doctor’s advice [40]. However, no significant differences were observed between the CD and UC groups in the level of satisfaction with the quality of IBD care received in the past 12 months. Female patients regarded the quality of IBD care as being worse than male patients. This can be attributed to several factors. A large population-based cohort showed no significant differences in terms of HRQoL between patients with UC and those with CD, overall, whereas a significant reduction in HRQoL was observed in women but not in men compared to the background population [23]. Female patients with IBD show a larger number of intense concerns, a greater level of psychological disturbance, a higher symptom load, and a poorer QoL than men, resulting in reduced satisfaction ratings [41–44]. A lower level of satisfaction amongst female patients was also noted in the QUOTE-IBD studies [45, 46]. Moreover, the finding that indicates a lower level of satisfaction amongst women is consistent with those of many other patient satisfaction-related studies [47, 48]. Secondly, no statistically significant difference was observed between the CD and UC groups in the assessment of the level of communication with health specialists in the past 12 months; however, a greater number of female than male patients assessed the level of communication as being worse. This finding suggests that, in the formulation of an appropriate communication strategy and provision of information about the disease, one should take into account subjective perceptions of QoL and sex-related differences [49]. In terms of the influence of demographic and clinical data on the level of satisfaction with the quality of IBD care in the current study, female patients, patients with significant pain or discomfort, those who felt a lack of energy or were fatigued for the majority of the time, and patients who experienced anxiety or depression in the 2 weeks preceding questionnaire administration were found to be less satisfied with the quality of IBD care than their counterparts. Similarly, a previous study also showed that anxiety-related symptoms were determinants of patient satisfaction among youths/adolescents with IBD [50]. In our study, no major differences related to the patient-perceived QoC was noted when stratification was performed according to disease subtype, age, disease duration, education level or abdominal surgery history. Nonetheless, a previous study showed that older age was associated with modestly higher scores on the Short Inflammatory Bowel Disease Questionnaire and mental HRQoL, but a lower physical HRQoL score [51]. Another study using PROMIS instruments showed that patients with CD had a significantly worse degree of impairment than those with UC in a population of ambulatory adults [49]. However, a prospective study conducted in a high-volume IBD center proved that perceived QoC is associated with sex, disease activity, work productivity and QoL, but not disease phenotype [52].

The bias and difference between specialists and patients requires investigation. Specialists should strive to improve the quality of doctor-patient communication. The differences in the scoring trends between physicians and patients could be related to the generally insufficient communication status due to high inpatient and outpatient department volumes and relative scarcity of healthcare providers, as well as the higher expectations of patients receiving IBD care at centers in first-tier cities. Furthermore, half of the patients considered the consultation time to be insufficient. Moreover, only two-thirds of the patients had a regular IBD review appointment regardless of the IBD activity status. Most patients were used to seeking specialist advice during a flare-up at hospitals or clinics; few received advice through telephone or email. The government should allocate a larger volume of funds aimed at improving the doctor-to-patient ratio to ensure better communication between physicians and patients.

Furthermore, in this era of growing healthcare cost constraints, providers must also demonstrate a favorable cost:benefit ratio with patient-centered outcome-based intervention; this requires further evaluation beyond the scope of this study. However, making improvements to some of the patient-centered outcomes (for instance, those items related to communication, information, consultation topics, and staff availability) may only result in small or negligible costs.

This study has some limitations. Firstly, the participants were enrolled from care centers across the eastern parts of mainland China, and may not be representative of the entire Chinese IBD population. The regions included in our study are more developed and industrialized than other parts of China. Secondly, the current study had a cross-sectional observational design. We cannot infer causality from these results, although it was possible to identify measures for improvements in clinical practice and patient care. Last, but not least, this study included a high number of statistical tests, which may result in type I errors. Despite these limitations, the current study provides important insights into the factors associated with patient-centered outcomes, as well as broadens the scope of the discussion pertaining to the status of IBD care in China.

In conclusion, we found that both patients and physicians perceived the IBD-related QoC as being satisfactory. We also identified areas of shortcomings and improvement where it comes to patient perception, and established that female sex and the presence of certain negatively impacting disease characteristics were associated with lower satisfaction levels. Therefore, patient concerns pertaining to their disease must be reassessed regularly, since there are certain aspects of IBD-care that may be improved, as evident from this study.

## Supplementary information

**Additional file 1:Table 1.** Demographics

**Additional file 2: Table 2.** Clinician Questionnaire

## Data Availability

The datasets collected and/or analyzed during the current study are available from the corresponding author upon reasonable request.

## Abbreviations

QoC: Quality of care
QoL: Quality of life
IBD: Inflammatory bowel disease
CD: Crohn’s disease
HRQoL: Health-related QoL
UC: Ulcerative colitis
IBD-U: IBD unclassified
CI: Confidence interval
