# Supplementary material for: Assessment of patient-centered outcomes (PROs) in inflammatory bowel disease (IBD): a multicenter survey preceding a cross-disciplinary (functional) consensus
Source: Health Qual Life Outcomes. 2020 Jul 20;18:241. doi: 10.1186/s12955-020-01489-8 (PMC7372780; doi:10.1186/s12955-020-01489-8)
Supplement: Supplementary file 1 — Additional file 1:Table 1. Demographics [file 12955_2020_1489_MOESM1_ESM.docx]

**Demographics**

| **1** | **Have you been diagnosed with** |  |
| --- | --- | --- |
|  | Ulcerative Colitis |  |
|  | Crohns Disease |  |
|  | Unspecified IBD |  |
| **2** | **Are you male or female?** |  |
|  | Male |  |
|  | Female |  |
|  |  |  |
| **3** | **What was your year of birth?** |  |
|  |  |  |
| **4** | **In what year were you diagnosed with IBD?** |  |
|  |  |  |
| **5** | **What is your highest level of education?** |  |
|  | Primary education |  |
|  | Secondary education |  |
|  | University education |  |
|  |  |  |
| **6** | **What is your current occupation?** |  |
|  | Working (employee) |  |
|  | Working (self-employed) |  |
|  | Studying |  |
|  | Unemployed |  |
|  | Long-term sick leave because of IBD |  |
|  | Long-term sick leave for other reasons |  |
|  | Retired early due to IBD |  |
|  | Retired early for other reasons |  |
|  | Retired (because of age) |  |
|  | Fulfilling family role as parent or partner |  |
|  |  |  |
| **7** | **Are you currently a smoker?** | **Yes/ No** |
|  | If yes, every day or almost every day |  |
|  | At least once per week (but not everyday) |  |
|  | 1  At least once per month (but not every week) |  |
|  |  |  |
| **8** | **Have you had abdominal surgery for IBD?** | **Yes/ No** |

**9-11**

**About your IBD in the last two weeks**

| **9** | **Do you believe that your IBD has been well controlled in the last two weeks?** |  |
| --- | --- | --- |
|  | Yes |  |
|  | No |  |
|  | Not sure |  |
|  |  |  |
| **10** | **Do you believe that your current treatment is useful in controlling your IBD?** |  |
|  | Yes |  |
|  | No |  |
|  | Not sure |  |
|  | I am not currently taking any treatment |  |
|  |  |  |
| **11** | ***In the past two weeks did you:*** |  |
| **a** | **Miss any planned activities because of your IBD?** |  |
|  | Yes |  |
|  | No |  |
|  | Not sure |  |
| **b** | **Wake up at night because of symptoms of IBD?** |  |
|  | Yes |  |
|  | No |  |
|  | Not sure |  |
| **c** | **Suffer from significant pain or discomfort?** |  |
|  | Yes |  |
|  | No |  |
|  | Not sure |  |
| **d** | **Often feel lacking in energy fatigued? (By often we mean more than half the time)** |  |
|  | Yes |  |
|  | No |  |
|  | Not sure |  |
| **e** | **Feel anxious or depressed because of your IBD?** |  |
|  | Yes |  |
|  | No |  |
|  | Not sure |  |
| **f** | **Think you needed a change to your treatment?** |  |
|  | Yes |  |
|  | No |  |
|  | Not sure |  |

**Your IBD Care before Diagnosis**

| **12** | **From the time of first seeking medical care for symptoms that you now recognise as related to IBD, how long did it take to get a confirmed IBD diagnosis? (months)** |  |
| --- | --- | --- |
| **13** | **Thinking back, how many times did you go to an emergency department or emergency clinic with IBD symptoms before you received a definitive diagnosis?** |  |

**The recent impact of IBD on your life**

| **14** | **How many flare-ups have you experienced in the last 12 months? If you are not sure, please give your best estimate.** |  |
| --- | --- | --- |
| **15** | **How many times have you been admitted to hospital because of IBD-related symptoms in the last 12 months? If you are not sure, please give your best estimate.** |  |
| **16** | ***How many days in the last 12 months have you...*** |  |
| **a** | **been absent from school or college due to IBD-related symptoms?** |  |
| **b** | **been absent from work due to IBD-related symptoms** |  |
| **c** | **been unable to do normal activities, due to IBD-related symptoms?** |  |
| **17** | **In the last 24 months, what is the longest continuous stretch of time that you have been taking steroid tablets for your IBD?**  **(months)** |  |

**Your experience of IBD Care**

| **18** | **Who do you normally consult about your IBD?** |  |
| --- | --- | --- |
|  | /GP/Family doctor |  |
|  | Gastroenterologist |  |
|  | General surgeon |  |
|  | Colorectal surgeon |  |
|  | Other hospital doctor |  |
|  | Specialist nurse |  |
|  |  |  |
| **19** | **Do you have a regular IBD review appointment whether or not your IBD is active at the time?** |  |
|  | Yes |  |
|  | No |  |
|  |  |  |
| **20** | **How long did your last IBD review consultation last? (minutes)** |  |
|  |  |  |
|  |  |  |
|  |  |  |
| **21** | **Did you consider this was enough time to review your IBD satisfactorily?** |  |
|  | Yes |  |
|  | No |  |
|  |  |  |
| **22** | **Who do you normally get your specialist advice from if you have a flare up?** |  |
|  | / GP/Family doctor |  |
|  | Gastroenterologist |  |
|  | General surgeon |  |
|  | Colorectal surgeon |  |
|  | Other hospital doctor |  |
|  | Specialist nurse |  |
|  |  |  |
|  | I have no access to specialist advice in case of a flare-up |  |
|  |  |  |
| **23** | **How do you usually get your specialist advice if you have a flare-up?** |  |
|  | Telephone |  |
|  | Hospital or clinic |  |
|  | Email |  |
|  |  |  |
| **24** | **In what year did you last have an overnight stay in hospital in relation to your IBD?** |  |
|  |  |  |
| **25** | **On that occasion, were you on a specialist gastroenterology ward?** |  |
|  | Yes |  |
|  | No |  |
|  | Don’t know |  |
|  |  |  |
| **26** | **On that occasion, did you think that the number of toilets was adequate for a patient with IBD?** |  |
|  | Yes |  |
|  | No |  |
|  |  |  |
| **27** | **Overall, do you feel that your IBD management is well coordinated?**  ***(For example: tests are arranged in a coordinated way, you can see different specialists in the same visit and do not need to come to the hospital on several occasions, etc.)*** |  |
|  | Yes, very well coordinated. |  |
|  | Yes, fairly well coordinated |  |
|  | No, fairly uncoordinated |  |
|  | No completely uncoordinated |  |
|  |  |  |

| **28** | **Overall, how would you rate the way the health specialists involved in your IBD care communicate with you?** | | | |  |
| --- | --- | --- | --- | --- | --- |
|  | Excellent | | | |  |
|  | Very good | | | |  |
|  | Good | | | |  |
|  | Fair | | | |  |
|  | Poor | | | |  |
|  |  | | | |  |
| **29** | **Overall, how do you rate the quality of the IBD care that you have received in the past 12 months?** | | | |  |
|  | Excellent | | | |  |
|  | Very good | | | |  |
|  | Good | | | |  |
|  | Fair | | | |  |
|  | Poor | | | |  |
|  | I did not receive any IBD care in the last twelve months. | | | |  |
|  |  | | | |  |
| **30** | **Thinking about your IBD consultations in the last 12 months, overall, have these topics been sufficiently discussed for your needs?** | | | |  |
|  |  | **Yes** | **No** | **No need to discuss** | **Don’t know** |
| **a** | **Current symptoms** |  |  |  |  |
| **b** | **Medical treatments** |  |  |  |  |
| **c** | **Surgery** |  |  |  |  |
| **d** | **New/experimental treatments** |  |  |  |  |
| **e** | **Nutrition/diet** |  |  |  |  |
| **f** | **Practical daily living** |  |  |  |  |
| **g** | **Education/studies** |  |  |  |  |
| **h** | **Employment** |  |  |  |  |
| **j** | **Personal relationships** |  |  |  |  |
| **k** | **Sexual relationships** |  |  |  |  |
| **l** | **General lifestyle issues** |  |  |  |  |
|  |  |  |  |  |  |
| **31** | **How often, after your IBD consultations in the last 12 months, did you feel you did not get to share something about your IBD that may have been important?** | | | |  |
|  | Always | | | |  |
|  | Most of the time | | | |  |
|  | Much of the time | | | |  |
|  | Sometimes | | | |  |
|  | /Hardly ever/never | | | |  |
|  | Not applicable/Don’t know | | | |  |
|  |  | | | |  |

| **32** | **During the past 12 months, when you received care, has any healthcare professional you see for your IBD** | | | |  |
| --- | --- | --- | --- | --- | --- |
|  |  | | **Yes** | **No** | **Don’t know** |
|  | ***discussed with you your main goals or priorities in caring for your condition?*** |  |  |  |  |
|  | ***helped you make a plan that you could carry out in your daily life?*** |  |  |  |  |
|  |  | | | |  |

**33. What for you is the best thing about the care you currently receive?**

**34. What is the one thing you would most like to see changed and improved?**

**THANK YOU FOR HELPING US WITH THIS SURVEY**
