# Supplementary material for: Assessment of patient-centered outcomes (PROs) in inflammatory bowel disease (IBD): a multicenter survey preceding a cross-disciplinary (functional) consensus
Source: Health Qual Life Outcomes. 2020 Jul 20;18:241. doi: 10.1186/s12955-020-01489-8 (PMC7372780; doi:10.1186/s12955-020-01489-8)
Supplement: Supplementary file 2 — Additional file 2: Table 2. Clinician Questionnaire [file 12955_2020_1489_MOESM2_ESM.docx]

**Clinician Questionnaire**

1. Are you male or female?
2. How many years have you been involved in caring for IBD patients?
3. What % of your work time involves caring for IBD patients?
4. Overall, do you feel that your IBD management is well coordinated? *(For example: tests are arranged in a coordinated way, patients can see different specialists in the same visit and do not need to come to the hospital on several occasions, etc.)*

Yes, very well coordinated./ Yes, fairly well coordinated./ No, fairly uncoordinated./ No completely uncoordinated.

5. Overall, how would you rate your communication with your patients in IBD care?

Excellent/ Very good/ Good/ Fair/ Poor.

6. Overall, how do you rate the quality of the IBD care that you have offered in the past 12 months?

Excellent/ Very good/ Good/ Fair/ Poor.

7. Thinking about your IBD consultations in the last 12 months, overall, have these topics been sufficiently discussed?

|  |  | Yes | No | No need to discuss | Don’t know |
| --- | --- | --- | --- | --- | --- |
| a | Current symptoms |  |  |  |  |
| b | Medical treatments |  |  |  |  |
| c | Surgery |  |  |  |  |
| d | New/experimental treatments |  |  |  |  |
| e | Nutrition/diet |  |  |  |  |
| f | Practical daily living |  |  |  |  |
| g | Education/studies |  |  |  |  |
| h | Employment |  |  |  |  |
| j | Personal relationships |  |  |  |  |
| k | Sexual relationships |  |  |  |  |
| l | General lifestyle issues |  |  |  |  |

8. During the past 12 months, have you…?

|  | Yes | No | Don’t know |
| --- | --- | --- | --- |
| *discussed with you patient his main goals or priorities in caring for his condition?* |  |  |  |
| *helped him make a plan that he could carry out in his daily life?* |  |  |  |
